# Exosomes may be the carrier of acupuncture treatment for major depressive disorder

**DOI:** 10.3389/fnbeh.2023.1107265

**Published:** 2023-02-16

**Authors:** Qin Lyu, Xin Zhou, Liu-Qing Shi, Hai-Yang Chen, Mei Lu, Xian-De Ma, Lu Ren

**Affiliations:** ^1^Graduate School, Liaoning University of Traditional Chinese Medicine, Shenyang, China; ^2^Department of Acupuncture and Moxibustion, Liaoning University of Traditional Chinese Medicine, Shenyang, China; ^3^Teaching and Experiment Center, Liaoning University of Traditional Chinese Medicine, Shenyang, China

**Keywords:** acupuncture, exosomes, major depressive disorder, Traditional Chinese Medicine (TCM), alternative therapies, opportunity, mechanism, antidepressant

## Abstract

The incidence of major depressive disorder (MDD) is increasing all over the world. There is a great need for complementary or alternative therapies with high safety, few side effects, and precise efficacy to care for MDD. In China, acupuncture has significant laboratory data and clinical trials to demonstrate its antidepressant efficacy. However, there is no clear answer as to how it works. Exosomes are membranous vesicles that rely on cellular multivesicular bodies (MVBs) fused to the cell membrane for release into the extracellular matrix. Almost all cell types are capable of producing and releasing exosomes. As a result, exosomes contain complex RNAs and proteins from their relatives (Cells that secretes exosomes). They can cross biological barriers and participate in biological activities, such as cell migration, angiogenesis, and immune regulation. These properties have made them a popular research topic. Some experts have suggested that exosomes may serve as delivery vehicles for acupuncture to work. This presents both an opportunity and a new challenge for improving the protocols of acupuncture as a treatment for MDD. To better define the relationship between MDD, exosomes, and acupuncture, we reviewed the literature from the last few years. Inclusion criteria included randomized controlled trials and basic trials evaluating acupuncture in the treatment or prevention of MDD, the role of exosomes in the development and progression of MDD, and the role of exosomes in acupuncture. We believe that acupuncture may affect the distribution of exosomes *in vivo*, and exosomes may be a new carrier for acupuncture treatment of MDD in the future.

## 1. Introduction

**FIGURE 1:**
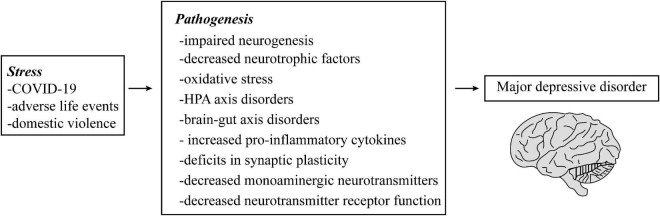
The possible pathogenesis of major depressive disorder (MDD).

Major depressive disorder is an affective disorder mainly characterized by depressive mood (Malhi and Mann, 2018). It is also a heterogeneous condition with different clinical presentations, severity, and longitudinal course (Herrman et al., 2022), such as depressed mood, slowed thinking, and reduced volitional behavior (Tandon, 2014). Its pathogenesis is influenced by multiple factors, such as genetic (Feurer et al., 2022), environmental (van den Bosch and Meyer-Lindenberg, 2019), and psychological (Fu et al., 2021). As neuronal activity is governed by synaptic inputs from excitatory or inhibitory neural circuits, the impairment of neural circuits involved in emotion, cognition (Price and Drevets, 2012), and synaptic plasticity (Duman et al., 2016) is often considered as the “main culprit” for the development of MDD. For example, some studies have found that MDD patients have overactivation of the amygdala and the prefrontal cortex, which are involved in the processing of negative emotions. This results in poor cognitive regulation of emotions and a tendency to process negative information related to the self (Seo and Oemisch, 2020; Asede et al., 2022; Rolls et al., 2023). The hippocampus, the most central region responsible for learning, emotion, and cognitive functions, has decreased density, reduced dendrites, and neuronal necrosis. Atrophy of the hippocampal region directly corresponds to memory loss, cognitive decline, and depressed mood states (Sheline et al., 2019; Du et al., 2021). In contrast, hippocampal synaptic remodeling is mainly dependent on rapid mitochondrial bursting and fission processes (Divakaruni et al., 2018). As well as it requires continuous mitochondrial production of ATP and Ca^2+^ (Cardanho-Ramos and Morais, 2021)and high sensitivity to mitochondrial transport damage (Tseng et al., 2015; Course et al., 2017). This mechanism of occurrence may be the result of the activation of several signaling cascades. Among them, adenosine monophosphate-activated protein kinase (AMPK) is an important protein kinase for the sensory energy state of the cell and is the master switch for cellular energy metabolism. AMPK activity is susceptible to stress (Hardie et al., 2012; Grahame Hardie, 2014). It has been clearly shown that AMPK activity reduction is involved in the control of depressive behavior (Odaira et al., 2019; Nakagawasai et al., 2020). Moreover, the aging process of organisms is often accompanied by a downregulation of AMPK activity, which tends to be higher in young animals than in older ones (Burkewitz et al., 2014). This explains to some extent why menopausal populations are more likely to suffer from MDD. Mitochondrial damage may be a common theme of research in other psychiatric disorders. In addition, MDD patients also have abnormalities in the neuro-endocrine-immune network system, particularly activation of the neuroinflammatory response and hyperactivity of the hypothalamic-pituitary-adrenal (HPA) axis, with crosstalk between them (Zunszain et al., 2011; Kim et al., 2016). Immune cells, such as monocytes, macrophages, lymphocytes, and dendritic cells, can secrete a series of pro-inflammatory cytokines interleukin-6 (IL-6), IL-1β, and tumor necrosis factor-α (TNF-α), which affects the neural circuitry of emotion and cognition. These cytokines are involved in the initiation, recurrence, and progression of MDD (Young et al., 2014; Adzic et al., 2018). Besides, many other pathogenic hypotheses have been proposed, such as impaired neurogenesis (Du Preez et al., 2022), decreased neurotrophic factors (Lee et al., 2022), oxidative stress (Bhatt et al., 2020), brain-gut axis disorders (Gheorghe et al., 2022), and decreased monoaminergic neurotransmitters (Joca et al., 2015); triggering one of these may cause a cascade of responses affecting the cerebral cortex (Figure 1).

However, suitable and satisfactory cures for these cascade problems have not been found in clinical practice. Not only that, but the rate of relapse and comorbidity of MDD is increasing. In 2008, the World Health Organization (WHO) listed MDD as the third most burdensome disease in the world and estimated that it will rank first by 2030 (Walton et al., 2006). Plus, with the spread of the COVID-19 pandemic, the likelihood of suffering from MDD has increased significantly in different occupational groups (Lokman and Bockting, 2022; Steen et al., 2022), such as COVID-19 survivors (Mazza et al., 2022), healthcare workers (Aoun et al., 2021; Moro et al., 2022), and self-quarantined people (Nantaayi et al., 2022) around the world. Besides, MDD occurs not only as a primary disease alone, but also merges with other underlying diseases, including perimenopause (Rudzinskas et al., 2021), puerperium (Zeng et al., 2022), stroke (Zhou et al., 2022), diabetes (Bakker et al., 2022), Parkinson’s disease (Xie et al., 2022), epilepsy (Aimaier et al., 2022), hepatitis (Gupta et al., 2020), gastritis (Eustis et al., 2022), and brain cancer (Nazli and Sevindik, 2022), as well as promotes the pathological development of the above diseases in turn. Thus, its occult pathogenesis features can easily lead to misdiagnosis (Kostaras et al., 2017). There are also adverse reactions to antidepressants (Uher et al., 2009), low patient compliance (Khalifeh and Hamdan-Mansour, 2021), withdrawal symptoms (Kendrick, 2021), drug resistance (Touloumis, 2021), and many other issues, which force us to find supplementary or alternative therapies with high safety, few side effects, and accurate curative effects.

Acupuncture has good efficacy and few side effects in the treatment of MDD (Armour et al., 2019; Yang et al., 2022). This can be strongly evidenced by the regulation of neurotransmitters, intestinal flora (Li P. et al., 2021), neuroplasticity (Li X. et al., 2020), inflammation (Kwon et al., 2012), and other molecular mechanisms, all of which explain its antidepressant effect. Acupuncture has a history of thousands of years. Its operating principle is to correct the imbalance of yin and yang in the body by piercing fine needles into specific acupoints according to the theory of the “eight-principles” and the concepts of “Zang-fu” and “meridians and collaterals” syndrome differentiation. However, many principles are based on traditional Chinese anatomy and philosophy, rather than Western neurophysiology and anatomy, so it is difficult to integrate Chinese and Western medicine and limit the transformation of basic research on acupuncture into modern medicine (Smith et al., 2018). In recent years, exosomes have received much more attention, and current modern biological studies have begun to analyze their ability to assess the effectiveness of acupuncture for several diseases, such as emphysema (Zou et al., 2021), ischemic stroke (Zhang et al., 2020), and kidney disease (Klein and Wang, 2018). At the same time, this knowledge may inspire acupuncture to explore the mechanism of prevention and treatment of MDD by using exosomes as an application carrier. Thus, we summarize the relationship between MDD, exosomes, and acupuncture to provide some enlightenment for the development and innovation of acupuncture to treat MDD.

## 2. The background of exosomes and MDD

**FIGURE 2:**
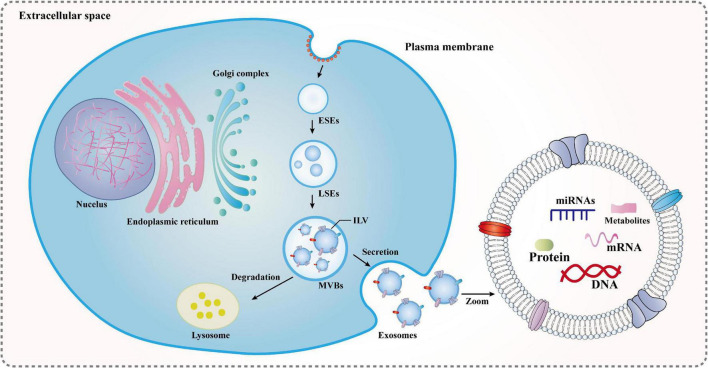
Formation mechanisms of exosomes: ESEs, early endosomes; LSEs, late endosomes; ILVs, intraluminal vesicles; and MVBs, multivesicular bodies. The plasma membrane undergoes invagination and fusion to form ESEs, and then ESEs continue to develop into mature LSEs and produce MVBs. MVBs are ILVs formed by the reinvagination of multiple LSEs, whose components come from endocytic and secretory (endoplasmic reticulum/Golgi) pathways into the lumen. Subsequently, a fraction of MVBs is transported to autophagosomes or lysosomes for degradation. The fusion of the remaining MVBs with the plasma membrane relies on exocytosis to release ILVs, which are then called exosomes.

As a type of extracellular vesicle (EV), exosomes were first discovered in the supernatant of sheep erythrocytes cultured *in vitro* in the 1980s and can be secreted by various cells. However, exosomes have been often overlooked in the past because they are a superfluous membrane protein that is released to regulate membrane function during cell maturation or they act as an organelle that scavenges cell debris and knocks out cell surface molecules. After extensive research, in the mid-1990s, exosome-borne cargo was found to be involved in important physiological and pathological links, such as cell migration and differentiation, promotion of angiogenesis, modulation of the immune reactions, remission of the inflammatory response, and antitumor invasion. These are closely related to reproductive development, immune regulation, cardiovascular diseases, and neurological diseases, which have attracted the attention of many researchers (Mashouri et al., 2019; Nabariya et al., 2020). Exosomes measure approximately 40–150 nm in diameter, are released into the extracellular milieu by almost all mammalian cells in normal/abnormal stages, and are found in body fluids, such as tears, nasal secretions, saliva, blood, cerebrospinal fluid, amniotic fluid, and urine. Initially, the plasma membrane undergoes invagination and fusion to form early endosomes (ESEs), and then ESEs continue to develop into mature late endosomes (LSEs) and produce multivesicular bodies (MVBs). MVBs are intraluminal vesicles (ILVs) formed by the reinvagination of multiple LSEs, whose components come from endocytic and secretory (endoplasmic reticulum/Golgi) pathways into the lumen. Subsequently, a fraction of MVBs is transported to autophagosomes or lysosomes for degradation. The fusion of the remaining MVBs with the plasma membrane relies on exocytosis to release ILVs, which are then called exosomes (Kalluri and LeBleu, 2020; Figure 2). Furthermore, exosomes are heterogeneous and targeted, and their rate, size, and composition are highly dependent on the parental cell (Zhang et al., 2019). In particular, the composition has specific proteins, lipids, mRNA, and microRNAs (miRNAs), all of which are associated with the parental cell. Thus, exosomes are considered a mini-version of the parental cells. These substances are wrapped in exosomes and rely on circulatory pathways to reach neighboring cells and distant target cells, making them important players in intercellular signaling. At the same time, they are not only a biomarker for diagnosing pathology and prognosis, but also an important carrier for gene and drug delivery. Exosomes have great potential for clinical application and the development of innovative treatments (Zhang et al., 2015; Meldolesi, 2018). Moreover, some studies have demonstrated that the heterogeneity and targeting of exosomes can reflect the characteristics of acupuncture and meridian induction (Chen et al., 2015; Li N. C. et al., 2021), which also provides great support for this paper.

## 3. The role of exosomes in the development, diagnosis, and treatment of MDD

Recently, several studies showed that exosomes are involved in pathological processes, such as neurogenesis (Wei et al., 2020), neuroinflammation (Brites and Fernandes, 2015), and other pathological processes. They can be used as a source of depression-specific markers for the diagnosis and treatment of MDD. Due to the prominent ability of exosomes to modulate intercellular communication and transport across the blood-brain barrier (BBB) to help cellular transmission, neurogenesis, and synaptic plasticity in the central nervous system (CNS), scholars’ interest in studying the relationship between exosomes and MDD has gradually increased.

### 3.1. Exosomes in the development of depression: Initiation and invasion in the brain

The bioactive proteins and microRNAs contained in exosomes can create a microenvironment that is conducive to the progression of MDD, thereby supporting the spread of other diseases. For example, insulin resistance not only leads to hyperglycemia but also promotes neuroinflammation, mitochondrial dysfunction, and neurogenesis, which are associated with human depression (Watson et al., 2021). Nasca et al. (2021) isolated brain-derived exosomes from the plasma of MDD patients by labeling L1 cell adhesion molecule^+^ (L1CAM^+^) and then found that brain-derived L1CAM^+^ exosomes contained more insulin receptor substrate 1 (IRS-1). Moreover, elevated levels of IRS-1 were associated with suicide and anhedonia. The reason could be the impaired transport of insulin across the BBB, leading to the inhibition of the neuronal insulin signaling pathway and the accumulation of IRS-1 in exosomes. It is believed that insulin resistance (IR) in the brain may not only be the result of the development of mood disorders, but also the starting point of the development of mood disorders. Kuwano et al. (2018) suggested that some proteins related to neuroinflammation and synaptic function can travel from brain neurons to the peripheral blood with the help of exosomes, which was experimentally demonstrated. The results showed that peripheral blood CD81 (neuron-derived exosomes) of MDD patients were indeed positively correlated with neuron-related blood biomarkers synaptophysin (SYP), tumor necrosis factor receptor (TNFR1), and interleukin 34 (IL-34), thus helping to objectively assess the severity of clinical depressive symptoms. Exosomes-derived proteins may play a role in initiation and invasion in the development of MDD.

In addition to specific proteins, microRNAs can also be packaged into exosomes to facilitate communication between neurons (Brites and Fernandes, 2015). Xian et al. (2022) found that the exosomes-derived miR-9-5p could transmit from neurons to microglia, resulting in microglial M1 polarization and neuronal reinjury. It also increased the release of proinflammatory cytokines, including IL-1β, IL-6, and tumor necrosis factor-alpha (TNF-α), thus exacerbating depressive symptoms in MDD mice. Fan et al. (2022) found that miR-146a-5p-containing exosomes derived from microglia could be a key factor in regulating hippocampal dentate gyrus (DG) neurogenesis *via* the krüppel-like factor 4/cyclin-dependent kinase like 5 (KLF 4/CDKL 5) pathway, and inhibited the proliferation and differentiation of NSCs during the pathogenesis of MDD. It can be considered a new crosstalk channel between glial cells and neurons. Luarte et al. (2017) also showed that astrocyte-derived miRNA-containing exosomes have potential regulatory effects on neurogenesis under stress conditions. Moreover, previous studies also demonstrated that the miRNA profiles of MDD rats were altered, when the expression levels of brain-derived neurotrophic factor (BDNF), tropomyosin receptor kinase B (TrkB), and synaptophysin 1 in serum exosomes were reduced in the serum exosomes of MDD rats. These differential expressions were related to the Mitogen-activated protein kinases (MAPK) pathway, Wnt pathway, and mammalian target of rapamycin (mTOR) pathway (Fang et al., 2020). It can be seen that the cargo carried by exosomes has the potential to initiate and slowly invade the emotional cognition field of the brain, thereby achieving the purpose of promoting the occurrence and development of MDD. Previously, Sakamoto et al. (2021) proposed a similar idea that alterations in circulating EVs might underlie social stress-induced behavior in mice. Chronic stress induces peripheral and intracerebral immune changes and inflammation, leading to neuropathies associated with psychiatric disorders such as MDD. Overall, although the above studies still require further exploration and validation, it is sufficient to indicate that exosomes carrying specific proteins and miRNAs are likely to be potential biomarkers to initiate and invade the emotional and cognitive domains of the brain. Ultimately, they can exacerbate the development of MDD.

### 3.2. Exosomes in the diagnosis and treatment of MDD: Enemies become friends

Despite various clinical interventions, including exercise, psychology, medication, etc., relapse of MDD remains very common. It was reported that MDD is more likely to recur after drug treatment is discontinued (Ebell, 2022). Moreover, MDD often accompanies various stages of other diseases. These diseases are latent and undiagnosed until the occurrence of some sort of physical injury leads to mortality or disability. Therefore, there is an urgent need for robust biomarkers that can accurately identify high-risk individuals or predict relapse, thereby helping to effectively guide diagnosis and treatment. However, it is important that exosomes are secreted from the early stage of MDD, and the secreted cargo can eventually produce different effects due to different sources, opening up a new way for the diagnosis and treatment of MDD.

Being a negative agent, or an “enemy,” is a promising diagnostic marker to inform the occurrence of MDD. Li L. D. et al. (2021) found that plasma exosomal has-miR-335-5p was significantly upregulated and plasma exosomal has-miR-1292-3p was significantly downregulated in patients with treatment-resistant depression (TRD). Go and KEGG analyses indicated that these changes were associated with postsynaptic density, axon formation, and cell growth signaling pathways. Liang et al. (2020) showed that serum exosome-derived miR-139-5p in patients with MDD could be used as a potential diagnostic biomarker.

As a positive agent, or a “friend,” the exosome-derived content provides a direction for therapy. Clinical trials and animal experiments by Wei et al. (2020) also found the specific overexpression of hsa-miR-139-5p in the blood exosomes of MDD patients. When exosome-derived miR-139-5p from the blood of MDD patients was injected into normal mice, it caused depression-like behaviors and hippocampal neurogenesis impairment. However, when exosomes from blood that was taken from healthy people who do not suffer from MDD and intranasal treatment of miR-139-5p antagomir was injected into CUMS mice, it rescued depressive-like behaviors, inhibited neural stem cell (NSC) proliferation, and neuronal differentiation. This demonstrates that miR-139-5p, a negative regulator of NSC proliferation and neuronal differentiation, plays a role in MDD neurogenesis. A study by Wang et al. (2021) showed that injection of plasma exosomes from MDD patients could significantly improve the depression-like behavior of LPS-induced depression in mice. The mechanism may be to transmit sigma-1 receptors (Sig-1R) to the CNS, thereby improving the inflammatory response in microglia. Li P. et al. (2021) found that natural killer (NK) cell-derived exosomes carrying miR-207 targeted the leucine-rich repeat (Tril) interactor of toll-like receptor 4 (TLR4), inhibited nuclear factor-kappa B (NF-κB) signaling in astrocytes, and reduced the release of pro-inflammatory cytokines (IL-1β, IL-6, and TNF-α), to attenuate depression-like symptoms in CMS mice. Guo et al. (2020) showed that there was a minor expression of miR-26a in MDD, but exosomes derived from bone marrow mesenchymal stem cells (BMSCs) could increase the expression of miR-26a in corticosterone-induced depressed rats. Besides, superoxide dismutase (SOD) levels were increased, and malondialdehyde (MDA), lactate dehydrogenase (LDH), TNF-α, and IL-1β levels were reduced. Exosomes also promoted the proliferation of hippocampal neurons and inhibited apoptosis, thereby improving the damage of hippocampal neurons in depressed rats. Pusic et al. (2019) demonstrated that during environmental enrichment, progressively increasing interferon-gamma stimulation of dendritic cell-produced exosomes (IFNγ-DC-Exos) significantly improved myelination and reduced oxidative stress and susceptibility to diffuse depression disorder *in vivo* and *in vitro*. Gelle et al. (2021) found that serum exosomes from MDD patients taking antidepressants could help BDNF pass the BBB, resulting in increased BDNF levels, decreased pro-BDNF levels, and improved neuronal plasticity in the CNS. These exosome-wrapped cargoes are both enemies and friends, which can not only become promising diagnostic markers but also provide certain information and direction for treatment (reference Table 1).

**TABLE 1:** The role of exosomes-derived cargoes in major depressive disorder (MDD).

Cargoes	Sample source	Expression	The effect of exosomes-derived cargoes change	References
L1CAM^+^, IRS-1	DD patients’ plasma exosomes	↑, ↑	These are associated with suicide and anhedonia.	Nasca et al., 2021
SYP, TNFR1, IL34	MDD patients’ peripheral blood exosomes	↑	These are important blood biomarkers for patients with MDD	Kuwano et al., 2018
miR-9-5p	MDD patients’ serum exosomes	↑	Promoted M1 polarization in microglia and enhanced the release of proinflammatory cytokines (IL-1β, IL-6, and TNF-α), and reactivated SOCS2-JAK/STAT3 pathways.	Xian et al., 2022
miR-146a-5p	CUMS rats’ serum exosomes	↑	Regulated hippocampal DG neurogenesis *via* the KLF4/CDKL5 pathway and inhibited the proliferation and differentiation of NSCs	Fan et al., 2022
Synaptotagmin 1, BDNF, TrkB,	CUMS rats’ serum exosomes	↓, ↓, ↓	By regulating the MAPK pathway, Wnt pathway, and mTOR pathway	Luarte et al., 2017
miR-335-5, miR-1292-3p	TRD patients’ plasma exosomes	↑, ↓	By regulating postsynaptic density, axon formation, and cell growth signaling pathways	Li L. D. et al., 2021
miR-139-5p	MDD patients’ serum exosomes	↑	Use as a potential diagnostic biomarker	Liang et al., 2020
miR-139-5p	MDD patients’ blood exosomes	↑	Tail vein injection in normal mice induced depressive-like behavior and hippocampal neurogenesis impairment.	Wei et al., 2020
	Healthy volunteers’ blood exosomes	–	Tail vein injection into CUMS mice (+ miR-139-5p antagomir) rescued depressive-like behaviors, inhibited NSC proliferation, and neuronal differentiation	
Sig-1R	MDD patients’ plasma exosomes	↑	Transmit Sig-1R to the CNS, and improve the inflammatory response in microglia.	Wang et al., 2021
miR-207	NK cells-produced exosomes	↑	miR-207 targets TLR4 interactor with leucine-rich repeats (Tril), inhibits NF-κB signaling in astrocytes, decreases the release of pro-inflammatory cytokines in CMS mice	Li X. et al., 2020
miR-26a	BMSCs-produced exosomes	↑	miR-26a-BMSCs-Exos promotes the corticosterone-induced depressed rats’ proliferation of hippocampal neurons and inhibits apoptosis, ↑SOD levels, ↓MDA, LDH, TNF-α, and IL-1β levels,	Guo et al., 2020
IFNγ	Dendritic cell-produced exosomes	↑	IFNγ-DC-Exos improves myelination and reduces oxidative stress and susceptibility to diffuse depression disorder *in vivo* and *in vitro*.	Pusic et al., 2019
–	MDD patients’ serum exosomes	–	Helps BDNF pass the BBB, ↑BDNF levels, ↓pro-BDNF levels, and improves neuronal plasticity in the CNS.	Gelle et al., 2021

## 4. Background role of acupuncture in MDD

Acupuncture is an integral part of Traditional Chinese Medicine (TCM). A significant number of clinical and basic research data have verified the safety and efficacy of acupuncture in the treatment of MDD and acupuncture has been recognized and widely applied in many countries. It can be used alone or in combination with drugs, especially when combined with antidepressants, not only to improve primary and secondary depressive symptoms but also to reduce the side effects of drug treatment (Xu M. M. et al., 2022). We have summarized the past decade of research on the possible mechanisms of acupuncture therapy (reference Table 2).

**TABLE 2:** The possible mechanism of the antidepressant effect of acupuncture.

Acupuncture points	Model	Species	Possible antidepressant mechanisms of acupuncture	References
LI4 and LR3	CUS	Male SD rats	↑Sucrose consumption, ↑horizontal and vertical movement, ↓glutamate, and ↓SNARE complex-related mRNA and proteins (VAMP1, VAMP2, VAMP7, SNAP25, and Syntaxin1)	Fan et al., 2016
LI4 and LR3	CUMS	Male SD rats	↑Sucrose consumption, ↑food intake, ↓latency, and ↑glial glutamate transporter EAAT2 in the hippocampus and PFC	Luo et al., 2017
LI4 and LR3	CUMS	Male SD rats	↑AMPA receptor-related mRNA and proteins (GluR1, GluR2, Stargazin, Pick1, SYP, PSD-95, and GAP-43)	Jiang et al., 2020
GV20, GV29, LI4, LR3+ Physostigmine	CUMS	Male SD rats	↑Body weight, ↑latency feeding time, ↑sucrose preference rate, ↑horizontal and vertical activity frequency, ↑grooming frequency, ↓ACh in the serum and prefrontal cortex, ↑AChE in the serum and prefrontal cortex, and ↑spine density and synaptic proteins in the PFC (BDNF, GluR2, PSD95, and synapsin I)	Gao et al., 2021
GV20 and GV29	CRS	Male SD rats	↓HMGB1 and Iba-1 in the hippocampus and ↓serum TNF-α	Chen et al., 2022
GV20 and PC6	CUMS	Male SD rats	↓NO, iNOS, COX-2, and PGE _2_ NF-κB expression in the hippocampus and prefrontal cortex.	Lu et al., 2017
GV20 and GV29	CUMS	Male SD rats	↑Bcl-2, GAP-43, p-ERK/ERK, and BDNF in the hippocampus and prefrontal cortex	Li et al., 2017
DU20, EX-HN3, BL23, BL20, BL18, BL15, Ren4	SI	Male SD rats	↓Plasmatic CORT, ↑plasmatic T and E_2_, and ↑hippocampal BDNF	Davila-Hernandez et al., 2021
DU23 and PC7	CUMS	Male SD rats	↓NO and cGMP pathway in the hippocampus and plasma (cGMP, NO, NR1, nNOS, iNOS, NR2A, and NR2B)	Huang et al., 2019
A: DU20; B: PC6, HT7; C: ST36, SP6, LR3	DD	Patients	↑GDNF in the serum	Sun et al., 2013
GV20, BL23, and SP6	PDD	KM female mice	↑T and E_2_ in serum, ↓FSH and LH in serum, ↑5-HT, DA, and NE in the brain, ↑hypothalamic nerve cells, ↓the patina, synapses of the nerve in the hypothalamus	Guo et al., 2019
KI10, LR8, LU8, and LR4	CRS	Male C57BL/6 mice	Induced hippocampus, cingulate cortex, motor cortex, and thalamus activity, ↑5-HT1A receptor expression in the hippocampus, cingulate cortex, motor cortex, and thalamus, and ↑5-HT1B receptor expression in the cingulate cortex, motor cortex, and the thalamus.	Lee et al., 2019
GV20	MDD	Patients	Altered amygdala resting-state functional connectivity	Duan et al., 2020
RN12, RN10, RN6, RN4, KL17, ST24, and Qipang	MDD	Patients	Modulated resting-state functional connectivity of the amygdala by rs-fMRI	Wang et al., 2016
RN12, RN10, RN6, RN4, KL17, ST24, and Qipang	MDD	Patients	Modulated corticostriatal reward/motivation circuits by rs-fMRI	Wang et al., 2017c
GV20	MDD	Patients	↑The activation level of left PFC by fNIR, and ↑the correlation between activation of left dorsolateral PFC and acupuncture during executive function	Zhang et al., 2021
GV20 and EX-HN3	CRS	Male SD rats	It is related to inflammatory pathways, amino acid metabolism, and, especially TNF signaling pathway, NF-κB signaling pathway, and Toll-like receptor signaling pathway.	Wang et al., 2017a
GV20 and EX-HN3	CRS	Male SD rats	Inhibited the innate immune response and inflammatory response.	Wang et al., 2017b

### 4.1. Regulating neuroplasticity

Chronic stress and depression-like behaviors have been implicated in impaired neuroplasticity, such as neuronal atrophy and synaptic loss in the medial prefrontal cortex (PFC) and hippocampus. However, Fan et al. (2016) found that electroacupuncture (EA) stimulation at Hegu (LI4), and Taichong (LR3) had the same effect as riluzole in improving neuroplasticity of chronic unpredictable stress (CUS) rats, including sucrose consumption, horizontal and vertical movement. Moreover, the level of glutamate and the synaptic soluble N-ethylmaleimide-sensitive factor attachment receptor (SNARE) complex-associated proteins and genes were all reduced [SNAP25, syntaxin1, vesicle-associated membrane protein 1 (VAMP1), VAMP2, and VAMP7]. Using the same acupoints, Luo et al. (2017) found that the antidepressant effect of EA may increase the level of glutamate transporter EAAT2 in glial cells. Jiang also found that LI4 and LR3 attenuated depression-like behaviors and protected the neuroplasticity of chronic unpredictable mild stress (CUMS) rats by upregulating the expression of α-amino-3-hydroxy-5-methyl-4-isoxazole-propionic AMPA receptor-related mRNA and proteins in the hippocampus, including GluR1, GluR2, Stargazin, Pick1, SYP, PSD-95, and GAP-43 (Jiang et al., 2020). Gao et al. (2021) observed that physostigmine could exacerbate depression-like symptoms in rats induced by CUMS. Instead, depression-like symptoms with body weight, latency feeding time, sucrose preference rate, horizontal and vertical activity frequency, and grooming frequency could be reversed thanks to EA stimulation at Baihui (GV20 or DU20), Yintang (GV29 or EX-HN3), LI4, and LR3 points. Furthermore, acetylcholine (ACh), acetylcholinesterase (AChE), spine density, and synaptic proteins [glutamate receptor 2 (GluR2), postsynaptic density protein 95 (PSD95), synapsin I] in the PFC were also reversed, resulting in an antidepressant effect.

### 4.2. Inhibiting stress-induced neuroinflammation

Neuroinflammation is an important factor in the development of depression. For example, repeated social stress activates microglia in the medial PFC *via* the innate immune receptor TLR2/4. This triggers the expression of the inflammation-associated cytokine IL-1 and the tumor necrosis factor TNF, leading to neuronal atrophy and impaired responses in the medial PFC (Nie et al., 2018). Acupuncture of GV20 and GV29 ameliorated neuroinflammation in chronic restraint stress (CRS)-induced rats. The reason was the reversal of the high expression of ionized calcium-binding adaptor 1 (Iba1), TNF-α, and mobility group B1 (HMGB1) in the hippocampus (Chen et al., 2022). Another study showed that acupuncture at GV20 and Neiguan (PC6) significantly reduced nitric oxide (NO), inducible nitric oxide synthase (iNOS), prostaglandin E2 (PGE2), and epocloidase 2 (COX-2) levels, and also inhibited activation of NF-κB activity in CUMS-induced rats. It was suggested that the antidepressant-like effect of acupuncture therapy may happen through the regulation of NF-κB in the brain region to inhibit the secretion of inflammatory mediators (Lu et al., 2017).

### 4.3. Regulating neurotrophic factors

Neurotrophic factors affect the morphology and physiological function of neurons, especially the reduction of BDNF can inhibit the growth of neurons, and hinder the formation and stability of neuronal synapses and long-term potentiation in the brain (Castren and Monteggia, 2021). A study by Li et al. (2017) found that EA of GV20 and GV29 prevented hippocampal apoptosis, reversed the decline in Bcl-2 and growth-associated protein (GAP-43) expression, activated downstream targets of neurotrophin signal-regulated kinase (ERK) phosphorylation in the hippocampus and PFC, and increased ERK phosphorylation/ERK and BDNF expression. This suggested that chronic stress depression is regulated by the ERK signaling pathway and is ameliorated by EA. Davila-Hernandez et al. (2021) indicated that acupuncture at Du20, Yintang (EX-HN3), Shenshu (BL23), Pishu (BL20), Ganshu (BL18), Xinshu (BL15), and Guanyuan (Ren4) exerted antidepressant-like effects by decreasing the express of corticosterone (CORT), increasing the level of testosterone (T) and estradiol (E_2_) in plasma, and enhancing the level of hippocampal BDNF in socially isolated male rats. Huang et al. (2019) found that acupuncture at Shangxing (DU23) and Daling (PC7) exerted antidepressant effects by mediating nitric oxide (NO) and cyclic guanosine monophosphate (cGMP) signaling pathways in the CUMS rat model. A clinical study by Sun et al. (2013) found that EA at DU20 and Zusanli (ST36) group and Taichong (LR3), Sanyinjiao (SP6), Neiguan (PC6), and Shenmen (HT7) group were as effective in treating MDD as the fluoxetine group, and that EA was more advantageous in regulating the production of glial cell-derived neurotrophic factor (GDNF).

### 4.4. Regulating monoamine transmitters

Some studies have shown that persistent stress or brain dysfunction can lead to MDD by reducing the concentration and activity of monoamine neurotransmitters, including dopamine, norepinephrine, epinephrine, serotonin, and histamine. Drugs that increase the activity of monoamine neurotransmitters in the CNS or the concentration of intersynaptic gaps have the effect of improving mood and treating MDD. EA also had this effect. Guo et al. (2019) showed that EA at GV20, BL23, and SP6 could improve the activity and memory of PDD mice, improve sex hormone disorders in serum (elevated T and E2, FSH and LH decreased), increase the levels of monoamine transmitters (5-HT, NE, DA) in brain tissue, and improve the morphological changes of the hypothalamus. Lee et al. (2019) found that acupuncture at KI10, LR8, LU8, and LR4 alleviated depressive-like behaviors in CRS mice by regulating the hippocampus, cingulate cortex, motor cortex, and thalamus activity (considered as the specific area where acupuncture works). Furthermore, the level of serotonin receptors (5-HT1A and 5-HT1B) was also increased in the above brain areas. It is believed that the acupuncture-induced brain nerve activity and the selected acupoints determine the signaling pathways in the MDD brain region.

Additionally, we were able to analyze the relationship between acupuncture and MDD through brain imaging and high-throughput sequencing techniques. Through resting-state functional magnetic resonance imaging (rs-fMRI), Duan et al. (2020) found that the abnormal amygdala network in MDD patients could be improved by EA stimulation of GV20. Through rs-fMR, Wang also found that acupuncture at Zhongwan (RN12), Xiawan (RN10), Qihai (RN6), Guanyuan (RN4), Shangqu (KL17), Huaroumen (ST24), and Qipang (extra-point) not only modulated resting-state functional connectivity of the amygdala in MDD patients by rs-fMRI (Wang et al., 2016), but also therapeutically modulated corticostriatal reward/motivation circuits (Wang et al., 2017c). Using functional near-infrared spectroscopy (fNIR), Zhang et al. (2021) found that acupuncture at GV20 tended to enhance the activation of the PFC in patients with severe depression symptoms. Moreover, there was a correlation between the activation of the left dorsolateral PFC and acupuncture during the execution of acupuncture. Wang et al. (2017a),b explored the transcriptomic study of acupuncture GV20 and EX-HN3 on the frontal cortex and hippocampus of CRS rats by RNase sequencing (RNA-seq) technology. The results indicated that the antidepressant mechanism is related to regulating the frontal cortex, amino acid metabolism, and inflammatory pathways, especially toll-like receptor signaling, TNF, and NF-B signaling. These are all associated with the suppression of hippocampal stress-induced activation of innate immune responses and inflammatory responses. The above illustrated the effectiveness of acupuncture in regulating MDD, which has great clinical development value.

## 5. The development of acupuncture in exosomes: Whether the cause can be cured

**FIGURE 3:**
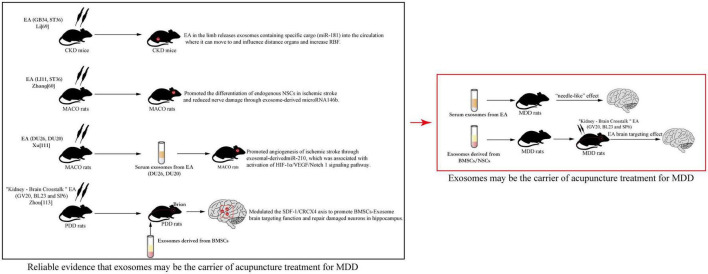
Reliable evidence that exosomes may be the carrier of acupuncture treatment for major depressive disorder (MDD).

Acupuncture has an incredible appeal: it is simple, convenient, inexpensive, clinically useful, and brings immediate effects. These features make acupuncture widely recognized and used worldwide. MDD is one of the specific diseases that can be treated by acupuncture. This form of treatment can be used alone or in combination with TCM and Western medicine to alleviate MDD (Li et al., 2019). However, acupuncture is a form of physiotherapy that is so limited in terms of innovative development that it is less advanced in terms of basic research and clinical practice when compared, for example, to natural drugs. Currently, with the increased interest in exosomes and the emergence of the latest medical evidence, scholars have noted that exosomes can serve as modern biological carriers for the transformation of acupuncture effects. Li N. C. et al. (2021) proposed that serum exosomes are expected to develop into “acupuncture network drugs.” The reason being that acupuncture has its own network biology, and injecting exosomes that have been intervened by acupuncture into the body would have a “needle-like” effect. It was shown that exosomes derived from live cells after acupuncture intervention had low immunogenicity and could carry various active ingredients, so there is a possibility of exosomes being developed as “acupuncture network drugs.” It was also reported that exosomes derived from mast cells at stimulated acupoints activated neuroimmunomodulation and participated in local network activity caused by acupoint stimulation (Chen et al., 2015, 2017). To the best of our knowledge, there are no studies that explore MDD treated by acupuncture from an exosomes perspective, but we can draw insight from a few other studies. Klein and Wang (2018) found that acupuncture with low-frequency electrical stimulation at GB34 and ST36 stimulation increased miR-181 levels of serum exosomes derived from mice with chronic kidney disease (CKD) and targeted suppression of angiotensinogen to raise renal blood flow (RBF). Other studies identified that EA (LI11 and ST36) could not only promote the differentiation of endogenous NSCs in ischemic stroke, but also reduced nerve damage through exosome-derived microRNA146b, providing essential new insights into the ability of acupuncture to exert neuromechanical regulation through brain-derived exosomes (Zhang et al., 2020). Similar studies also showed that EA at Renzhong (DU26) and DU20 treatment could promote angiogenesis of ischemic stroke through exosomal-derived miR-210, which was associated with activation of the HIF-1α/VEGF/Notch 1 signaling pathway (Xu S. Y. et al., 2022). In addition, exosomes have the potential for non-invasive therapy, such as acting as drug carriers, or stem cell delivery carriers (Peng et al., 2020). This suggests that, in the future, we can explore the modern mysteries of acupuncture from the perspective of exosomes to better serve clinical care and society. Our group has also made some scientific speculations based on the following: the proliferation and differentiation of BMSCs and NSCs may be a modern manifestation of the process of “kidney essence entering the marrow and brain” in Chinese medicine. The group obtained preliminary data from animal and cellular experiments. Zhou’s experiments showed that the improvement of depressive behavior in rats with perimenopausal depression disorder (PDD) model by “Kidney–Brain Crosstalk” EA (GV20, BL23, and SP6) may be due to the modulation of the SDF-1/CRCX4 axis to promote BMSCs-Exosomes brain targeting function and repair damaged neurons in the hippocampus (Zhou, 2020). Analysis of the above literature, especially Chen et al. (2015, 2017), Klein and Wang (2018), Zhang et al. (2020), Zhou (2020), Li N. C. et al. (2021), and Xu S. Y. et al. (2022) provided us with viable ideas that acupuncture can facilitate exosomes to a designated location to exert their healing effects. Exosomes may be one of the carriers of the therapeutic effect of acupuncture, exerting similar effects to acupuncture (Figure 3).

The experiments of Li and Zhong suggested that acupuncture therapy in the treatment of MDD may also alleviate depression by affecting the content and concentration of endogenous exosomes. It is further suggested by Xu’s experiments that serum exosomes collected by acupuncture in MDD patients or animals may also have a similar effect to acupuncture when reinfused into the body. Through Zhou’s experiments, it was suggested that MDD patients or animals can target exogenous NSCs-Exosomes to reach brain sites and amplify the effect of acupuncture. This provided the basis for the main theme of this article “Exosomes may be the carrier of acupuncture treatment for MDD.”

## 6. Limitations and future directions

Inevitably, there is a limitation to this paper. There are few papers on the current physiopathological role of exosomes in MDD and the regulation of exosomes by acupuncture to improve depressive behaviors, which fails to fully reveal the specific mechanism and application potential of acupuncture in the field of regulating exosomes in depression. On the other hand, there are also some limitations in the field. Exosomes samples are not suitable for clinical settings due to the lack of standardized methods for collecting and isolating exosomes, and their low recovery rate and purity. Therefore, the biggest challenge for researchers to overcome is still how to obtain high-quality exosomes and their inclusions, and how to efficiently translate them to clinical care. In addition, acupuncture research faces many difficulties. For example, how to further improve the objectivity of animal experiments, how to scientifically explain the physiological effects of acupuncture points, and how acupuncture facilitates the targeted transport of substances by exosomes. The levels of serum exosomes collected after acupuncture at specific acupuncture points are uncertain, and none of the specific mechanisms have been more thoroughly elucidated. Examples include mitochondrial function, AMPK mechanisms, and the role of NSC-derived exosomes in MDD. These may become the main areas of focus in the future.

## 7. Conclusion

In summary, exosomes act as both friends (diagnostic targets, drug carriers) and enemies (delivery of pathogenic factors) in the pathogenesis of MDD. On the other hand, we know that acupuncture is effective in improving MDD. It is also known that the relationship between exosomes and acupuncture is that exosomes may carry information about the effects of acupuncture points. The structure and content of exosomes reflect the concept of “Deqi” in the principles of acupuncture. At the same time, the heterogeneity and targeting of exosomes also reflect some characteristics of acupuncture, such as the identification of acupuncture points and meridian transmission. It is proposed that exosomes may be the vehicle for acupuncture treatment of MDD. Their contents may not only carry therapeutic targets, but also reveal the differences in tonicity and variety of acupuncture techniques, and the selection and combination of specific acupuncture points. The emergence of exosomes seems to provide a feasible way to fully simulate the therapeutic effects of acupuncture, assuming that exosomes can become not only the transmitters during acupuncture treatment, but also carriers of needle-like effects at the end of an acupuncture intervention. Therefore, exosomes are very interesting and promising research components.

## Author contributions

QL: responsible for writing and making. XZ and H-YC: responsible for making. L-QS and ML: responsible for searching relevant literature. X-DM: responsible for providing advice. LR: responsible for providing advice and funding. All authors contributed to the article and approved the submitted version.
